# Robotic mobile and mirror milling of large-scale complex structures

**DOI:** 10.1093/nsr/nwac188

**Published:** 2022-09-07

**Authors:** Zenghui Xie, Fugui Xie, Limin Zhu, Xin-Jun Liu

**Affiliations:** State Key Laboratory of Tribology in Advanced Equipment, Department of Mechanical Engineering (DME), Tsinghua University, China; Beijing Key Lab of Precision/Ultra-precision Manufacturing Equipments and Control, Tsinghua University, China; State Key Laboratory of Tribology in Advanced Equipment, Department of Mechanical Engineering (DME), Tsinghua University, China; Beijing Key Lab of Precision/Ultra-precision Manufacturing Equipments and Control, Tsinghua University, China; State Key Laboratory of Mechanical System and Vibration, School of Mechanical Engineering, Shanghai Jiao Tong University, China; State Key Laboratory of Tribology in Advanced Equipment, Department of Mechanical Engineering (DME), Tsinghua University, China; Beijing Key Lab of Precision/Ultra-precision Manufacturing Equipments and Control, Tsinghua University, China

## Abstract

Robotization, miniaturization and portability have become the new development trend of milling equipment for large-scale complex structures, and the robotic mobile and mirror milling technology has reached remarkable progress in practical production.

Large-scale complex structures, such as spacecraft cabins, aircraft composite skins and launch vehicle fuel tanks, are the core components of the equipment in aerospace, energy, shipbuilding and other fields. Their milling quality and efficiency are factors directly affecting the performance and productivity of spacecraft, aircraft and other key equipment. This kind of structure generally features large dimension, high accuracy, low structural rigidity and complex geometry. While machining these super-large and heavy-duty products, the technological processes of transport, disassembly and reassembly may not be allowed or feasible. The complex structural form and strict machining requirements bring severe challenges to the performance of milling equipment.

The manufacturing of these structures requires extremely large workspace as well as greater mobility and flexibility of the milling equipment due to the large structural dimension and complexity. Nowadays, the mainstream milling mode for these structures adopts large customized machine tools supplemented by manual assisted repair. For example, aircraft beams have a length of 2–8 m, with 2–4-mm-thick meshed ribs distributed thereon. Large bent alloy panels, which are the main parts of heavy launch vehicle fuel tanks, have a diameter of 9–11 m and an arc length of 5–8 m, with meshed ribs in the shape of an equilateral triangle inside. To complete the pocket milling of meshed ribs, large five-axis machine tools are usually adopted to carry out enveloping milling, but this kind of milling equipment has a high cost and poor flexibility. In particular, with the increase in the design dimensions of these structures, the strokes of traditional machine tools have gradually become insufficient to meet the milling requirements. Because there exist many extreme manufacturing technology bottlenecks in the development of ultra-large milling equipment, it is difficult to sustain the large-scale development trend of traditional machine tools. Large-scale spacecraft cabins have a diameter of 3–5 m and a length of 6–10 m, and each is surrounded by >1000 installation brackets. With respect to the global reference coordinate system, high form and position accuracy is required for the mounting surfaces of these brackets. To complete the milling of these bracket mounting surfaces, the strategy of ‘separate milling, manual assembly and repair’ is usually adopted. But in the machining process, the benchmark needs to be transformed many times, which results in a long production period, low accuracy and poor product quality. Therefore, it is urgent to explore innovative machining technology and equipment to mill such large-scale complex structures with high efficiency and high quality.

Against this background, the industry and academia have made a lot of useful explorations, achieving remarkable results and giving a lot of inspiration. Thanks to the recent advances in robotics, the mobile robot *in situ* machining mode is developing gradually. The portable robotic machining unit is mounted on the mobile platform, which improves the machining accessibility, environmental adaptability and manufacturing flexibility of the robot, so as to realize the *in situ* machining of large-scale structures, as shown in Fig. 1. The Fraunhofer Institute for Manufacturing Technology and Advanced Materials (IFAM) constructed a mobile machining system using a serial industrial robot mounted on an automated guided vehicle (AGV), successfully carrying out the drilling operation on a large aircraft wing with a length of 6 m [1]. The mobile robot adopts the open-type machining method, which improves the adaptability to different objects, reduces the cost of machining equipment and provides a new solution for the machining of large-scale complex structures. Due to the low stiffness, poor performance consistency and drastic changes in stiffness and accuracy in the workspace boundary, serial robots are only suitable for machining scenarios with low accuracy requirements or a small contact force, but not applicable to milling operations with complex interaction forces.

**Figure 1.**
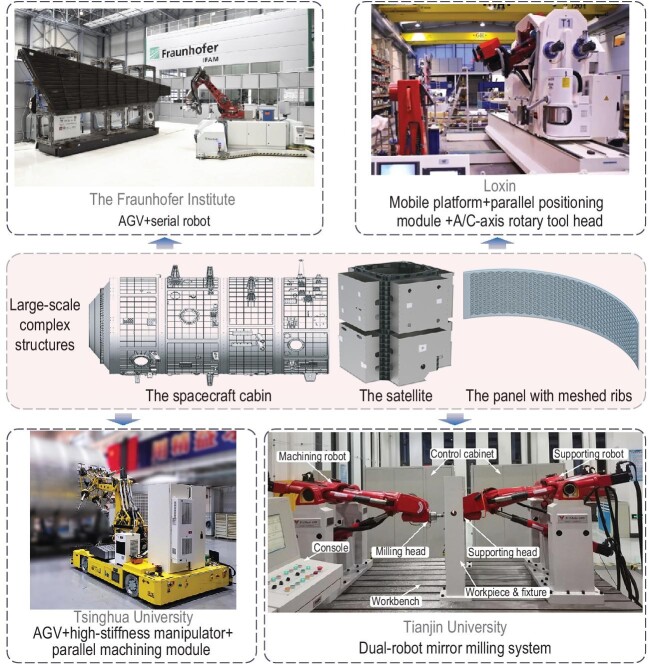
Robotic mobile and mirror milling. The upper-left picture is the mobile machining system developed by the Fraunhofer Institute. Adapted with permission from Ref. [1]. Copyright 2017, Elsevier. The upper-right picture is the mobile drilling robot developed by Loxin. Adapted with permission from Ref. [2]. Copyright 2019, Editorial Board of Journal of Mechanical Engineering. The lower-left picture is the mobile hybrid milling robot developed by Tsinghua University. The lower-right picture is the dual-robot mirror milling system developed by Tianjin University. Adapted with permission from Ref. [8]. Copyright 2018, Springer Nature.

The development of machining equipment is always accompanied by the innovation of mechanism principles and configuration. Parallel/hybrid robots have attracted extensive attention with their compact structure, high stiffness-to-mass ratio and excellent dynamic characteristics. Loxin, a company from Spain, developed a lightweight and modular five-axis hybrid machining robot Tricept by combining a three-DoF parallel positioning module and an A/C-axis rotary tool head [2]. In order to meet the machining requirements of large-scale structures, this hybrid robot is integrated with a mobile platform (i.e. a sliding table and a high-precision turntable) to form a mobile drilling robot, which has been successfully applied in the high-efficiency drilling of A350XWB aircraft panels. Tianjin University also developed a modular five-DoF hybrid robot TriMule by adopting the configuration of a ‘three-DoF parallel positioning mechanism + A/C-axis rotary tool head’ [3]. Integrated with an AGV, a mobile hybrid robot was developed, which has been successfully applied to mill the load mounting surfaces of large satellite structures. The above successful applications show that parallel robots can achieve high-efficiency and high-accuracy positioning through the coupling motion of multiple driving units. As a result, such mobile robots with parallel/hybrid robotic machining cores can be applied in milling operations with strong force interactions and high accuracy requirements. The only drawback is that the adjustment of the tool orientation is realized by the A/C-axis rotary tool head. There is a singular region in its orientation workspace and the large-range motion of the C-axis may occur when the cutter passes through the singular region [4], which may affect the machining efficiency, and even leave undesirable tool marks on the machined surfaces. This limits the improvement of machining equipment performance to a certain extent.

In order to break through the limitation of the traditional A/C-axis rotary tool head in tool orientation adjustment, DS-Technologie, a company from Germany, developed a parallel tool head named Sprint Z3, which has two rotational DoFs and one translational DoF [5]. Combined with a two-DoF sliding gantry, the five-axis hybrid machining center named Ecospeed is constructed. This machining center has been successfully applied in the manufacturing of aluminum alloy aircraft structural parts. Because Sprint Z3 achieves attitude adjustment through the coupling motion of three driving limbs and can adjust its tool orientation to an arbitrary direction directly and smoothly, the singular problem faced by the A/C-axis rotary tool head is solved, and the machining efficiency and accuracy of aircraft structural parts are improved greatly. This successful application demonstrates that parallel robots can achieve more efficient tool orientation adjustment through a multi-axis coupling motion.

Compared with the hybrid machining robot, the five-axis fully parallel robot has the potential of high dynamic response, modularization and miniaturization. Metrom, a company from Germany, developed a five-DoF parallel robot, which has been applied to machining of power plant components. Tsinghua University developed a five-axis fully parallel robot DiaRoM, which has the characteristics of coupling orientation adjustment and high-efficiency positioning [6]. Its driving units are all electric ball screws mounted between the base and the spindle, which facilitates the lightweight and modular design. Its performance specifics are listed as follows: the workspace is 600 × 600 × 400 mm^3^, the tilt angle can reach 30° in the Rotational Tool Center Point motion, the swing angle around the *X*-axis can reach 110°, the positioning accuracy is 0.018 mm and the weight of the parallel module is ∼800 kg. The production application of DiaRoM has been conducted in Chengdu Aircraft Industrial Corporation. The milling of key parts such as the frame-type structural part, cover plate, reinforced frame and junction plate is completed. The maximum milling error is 0.068 mm, which is far superior to the accuracy requirement for this kind of part.

Aiming at the *in situ* milling of large-scale structures, Tsinghua University further conducted the lightweight design for DiaRoM and developed a mobile hybrid robot with the configuration of ‘AGV + high-stiffness manipulator + lightweight five-axis parallel module’ [7]. This mobile milling robot named CraftsRobot, and has the ability of wide-range positioning and local high-accuracy milling. Its vertical stroke is 1650 mm, the workspace of the parallel module is 300 × 300 × 200 mm^3^, the positioning accuracy is 0.015 mm and the weight of the parallel module is only ∼300 kg. This robot has been successfully applied in practical production in Beijing Spacecrafts. (i) It completed the welding surface milling of a large spacecraft's entire cabin, and all of the products including four column sections and two cone sections are qualified (the milling accuracy is better than 0.1 mm). The milling period of a single column section and a single cone section is 10 and 6 h, respectively, which is better than the current milling scheme using a five-axis machine tool (column section 24 h, cone section 20 h). (ii) It completed the bracket mounting surface milling of a large spacecraft and the milling accuracy is better than 0.2 mm. In this way, the design concept of ‘anytime and anywhere’ five-axis milling is realized, which has disruptively changed the milling mode for large-scale spacecraft structures.

According to the above application cases, an ideal solution to milling large-scale complex structures is to construct a mobile hybrid robotic manufacturing system by integrating the *in situ* machining mode, the modular and lightweight design concept, and the parallel robots’ characteristics of coupling orientation adjustment and positioning. Such mobile hybrid robots featuring robotization, miniaturization and portability have gradually become the new development trend for milling equipment for large-scale complex structures.

Large-scale lightweight alloy skin panels are important parts that are used to shape aircraft, launch vehicle fuel tanks and manned spacecraft cabins. It is well recognized that the problems of deformation and vibration are quite prominent in the milling of extremely low-rigidity large-scale structural parts with a length-to-thickness ratio of >1000, so it is a great challenge to guarantee the thickness accuracy. For milling of such structures, it is necessary to introduce advanced machining processes. As a novel advanced machining process for high-efficiency and high-accuracy milling of large-scale lightweight skin panels, mirror milling technology and equipment have attracted extensive attention from industry and academia. Fruitful research has been carried out in this field and some companies have developed mirror milling equipment with excellent performance, such as the Dufieux Industrie from France, the M. Torres from Spain, the AVIC Manufacturing Technology Institute from China and TOP Numerical Control from China. These successful cases illustrate that mirror milling technology can improve the machining quality of thin-walled parts and stimulate us to promote this technology to the robotic milling field. To this end, Chinese researchers have developed a new technique for efficient and accurate milling of skin panels through a novel dual-robot mirror milling system, i.e. one robot takes charge of milling and error compensation, while the other cooperatively takes charge of mirror-following supporting and thickness measurement. Specifically, the end effectors of the milling robot and supporting robot implement symmetrical mirror movement along the two sides of the thin-walled part. The deformation and vibration during milling are suppressed through the variable stiffness control of the supporting robot's end effector. The wall thickness accuracy is guaranteed through real-time measurement and predictive control of the distance between the tool center point of the milling robot and the supporting point of the supporting robot. Tianjin University has developed a dual-robot mirror milling system based on two TriMule robots [8]; Shanghai Jiao Tong University has solved some key issues of dual-robot cooperative milling, such as the design of a shape-adaptive mirror-following supporting end effector with controllable stiffness, and the real-time closed-loop control of wall thickness [9,10], so that the wall thickness of large thin-walled structures is steadily controlled to within 1.5 ± 0.1 mm through compliant and precise dual-robot cooperative operation.

As a new round of global technological and industrial revolutions are in full swing, the manufacturing industry has seen further integration with electronic information, materials and other fields, which brings both opportunities and challenges to its high-end and intelligent development. Chinese scholars and enterprises have made bold attempts in the research and development of *in situ* manufacturing technology and equipment for large-scale complex structures, and have completed the verification and realized personalized machining application, adding momentum to the development of high-end and even intelligent manufacturing technology and equipment.

Featuring large dimensions and a multi-step machining process, the milling mode of large-scale complex structures has gradually transformed from ‘fixing the machine, moving the part’ to ‘fixing the part, moving the machine’. Multimodal perception and behavioral adaptation in robotic milling will become the frontier and hotspot of the future research, yet faces the following challenges:

***Integration of measurement and milling.*** The application of traditional perception is confined by the structured scene, the single mode and the limited perception space, so the measurement accuracy for oversized structures is limited. Therefore, it is necessary to break through the limitation of local perception in space and develop the ability of full scene perception; in this way, the robot's ‘sense of space’ and ‘global view’ for large-scale complex structures will be enhanced, and the integration of milling and measurement can be realized.

***Milling status real-time monitoring and control.*** Large-scale thin-walled structures have the characteristics of low rigidity, complicated structure and high accuracy requirements, which are extremely difficult to be manufactured. Due to the large dimension and thin thickness, random deformation and vibration usually occur in the milling process, which directly affect the milling accuracy. Therefore, the real-time monitoring and control of milling status under multi-sensor fusion is an essential and challenging step for quality guarantee.

***Multi-robot cooperative efficient milling*.** Traditional milling equipment mostly works in a standalone single-machine mode, which suffers from poor parallel cooperation ability and low efficiency. Therefore, it can be an ideal solution to break through the shortcomings of the single-machine mode from the perspective of working space and develop the multi-robot collaborative milling mode, then the ability of parallel collaborative operation and behavior adaptability for large-scale complex structures will be enhanced, and the milling efficiency can be improved.
